# Synergistic convergence and split pons in horizontal gaze palsy and progressive scoliosis in two sisters

**DOI:** 10.4103/0301-4738.77012

**Published:** 2011

**Authors:** Nitin R Jain, Jitendra Jethani, Kalpana Narendran, L Kanth

**Affiliations:** Department of Pediatric Ophthalmology and Strabismus, Aravind Eye Hospital, Coimbatore, India; 1 Dr. Thakorbhai V Patel Eye Institute, Vadodara, India; 2 Acura Scan Centre, Coimbatore, India

**Keywords:** Horizontal gaze palsy with progressive scoliosis, split pons sign, synergistic convergence

## Abstract

Synergistic convergence is an ocular motor anomaly where on attempted abduction or on attempted horizontal gaze, both the eyes converge. It has been related to peripheral causes such as congenital fibrosis of extraocular muscles (CFEOM), congenital cranial dysinnervation syndrome, ocular misinnervation or rarely central causes like horizontal gaze palsy with progressive scoliosis, brain stem dysplasia. We hereby report the occurrence of synergistic convergence in two sisters. Both of them also had kyphoscoliosis. Magnetic resonance imaging (MRI) brain and spine in both the patients showed signs of brain stem dysplasia (split pons sign) differing in degree (younger sister had more marked changes).

Synergistic convergence is a rare, abnormal extraocular muscle motility pattern consisting of bilateral adduction (convergence) during attempted lateral gaze in the absence of convergence spasm. Previously, three individual cases of synergistic convergence have been reported.[1–3] Horizontal gaze palsy with progressive scoliosis (HGPPS) is a known clinical entity, as a part of congenital cranial dysinnervation disorder syndrome (CCDDs).[4] Split pons (congenital cleavage of pons) in association with HGPPS has been reported rarely in literature.[5,6] To the best of our knowledge, this is the first case report of two sisters having synergistic convergence in association with kyphoscoliosis and split pons sign on magnetic resonance imaging (MRI).

## Case Reports

### Case 1

A 7-year-old girl was brought to the hospital with complaints of squinting since birth. Birth history and medical history were unremarkable. Her best corrected visual acuity (BCVA) in both the eyes was 20/40. Anterior segment and fundus examination was normal in both the eyes. In primary position, she had 30 prism diopter (PD) esotropia with mild left hypertropia. Examination of ocular movements revealed absence of abduction on either side. Rather, on attempted horizontal gaze to either side, both eyes adducted without pupillary miosis [Fig. 1a]. Vertical gaze was intact. Convergence to a near target was appropriate including pupillary miosis. Abduction (with and without monocular occlusion of other eye) could not be elicited. Forced duction and force generation test could not be performed as the child was uncooperative for the test.

**Figure 1a:**
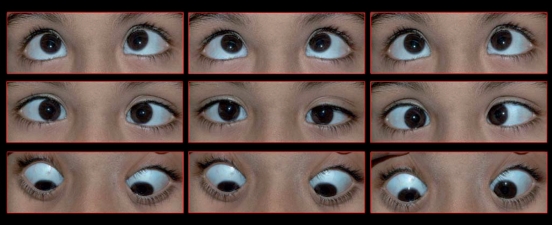
Clinical picture of case 1 showing nine gazes

Systemic examination revealed kyphoscoliosis. MRI brain and spine was performed, which showed abducens and oculomotor nerves with normal course and normal thickness. The extraocular muscles were normal. Deep midline pontine cleft (split pons sign) was seen. Kyphosis with convexity to the left was seen [Fig. 1b and c].

**Figure 1b:**
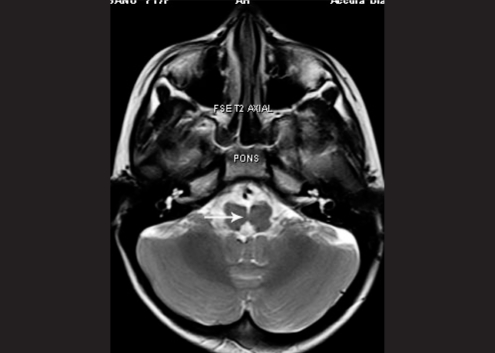
MRI brain of case 1, T2 axial, arrow denotes pontine cleft (split pons sign) (c) MRI spine of case 1, T2 coronal

**Figure 1c:**
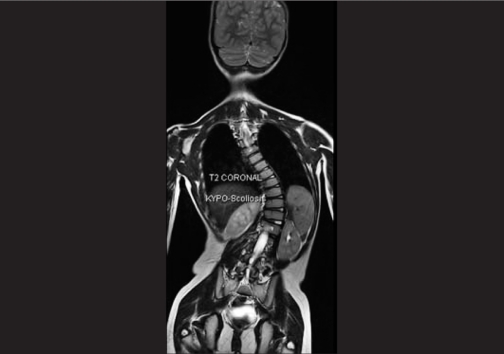
MRI spine of case 1, T2 coronal

### Case 2

A 14-year-old girl, who was the elder sister of the above-mentioned child, was brought to the hospital with complaints of abnormal eye movements since birth. Her birth history was unremarkable. Her BCVA in both eyes was 20/30. Anterior segment and fundus examination was normal in both the eyes. In primary position, she had 20 PD esotropia. Neither eye abducted on attempted horizontal gaze; rather, both the eyes adducted on attempted horizontal gaze [Fig. 2a]. Convergence to near target and pupillary reaction were normal. The child was uncooperative for force duction testing despite topical anesthesia, so it could not be performed. She had kyphoscoliosis, but of a milder degree as compared to her younger sister. MRI brain and spine was done. The extraocular muscles, abducens and oculomotor nerves were normal. Congenital cleavage or split pons sign was seen but the cleft was smaller compared to her younger sister. Spine showed kyphoscoliosis [Fig. 2b and c].

**Figure 2a:**
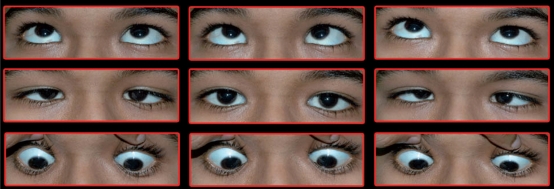
Clinical picture of case 2 showing nine gazes

**Figure 2b:**
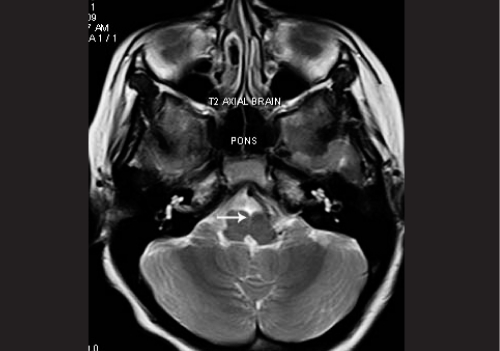
MRI brain of case 2, T2 axial, arrow denotes pontine cleft

**Figure 2c:**
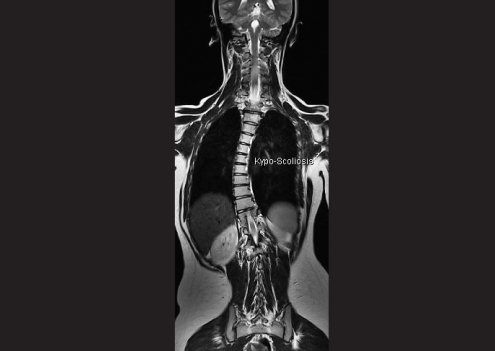
MRI spine of case 2, T2 coronal

As per the history given by the mother, the father of these children also had similar abnormal eye movements but he had expired few years back and therefore a definite evidence of the findings could not be presented.

## Discussion

Synergistic convergence[1–3] is an extremely rare form of ocular motor synkinesis characterized by simultaneous adduction on lateral gaze. Kim *et al*.[1] have described synergistic convergence in a patient who had congenital fibrosis syndrome and whose eye motility was limited in all directions in both the eyes.[1] MRI in that patient disclosed absence of the abducens nerves as well as hypoplasia of the oculomotor nerves with atrophy of the superior and medial rectus muscles of both the eyes.

In another case, Pieh *et al*.[2] described a child with synergistic convergence along with globe retraction and palpebral fissure changes.[2] MRI was normal in this patient. He described abducens nerve miswiring to the medial rectus as the cause for synergistic convergence. Khan *et al*.[3] described a case of synergistic convergence in association with kyphoscoliosis and brainstem hypoplasia. Family screening in this patient revealed both parents and both sisters as carriers and both brothers as non carriers. Bosley *et al*.[6] reported a patient with HGPPS with mutations in ROBO3.

Our patients differ from these two patients of synergistic convergence in that none of our patients had abnormality of abducens or oculomotor nerve and all the extraocular muscles were also normal. None of our patients had associated globe retraction or palpebral fissure changes. Moreover, the vertical ocular motility was normal. Ours is the first case in which more than one family member is affected. Their deceased father also had a similar clinical picture but definite evidence could not be obtained.

MRI findings in the case of HGPPS have been described. These include absence of facial colliculi, presence of deep midline pontine cleft (split pons sign), butterfly configuration of the medulla and kyphoscoliosis in spine.[5,6] None of the cases had split pons in synergistic convergence. Moreover, such findings in two sisters have not been reported.

Our two cases suggest that synergistic convergence could be a variant of HGPPS. One more interesting fact is that the degree of findings in MRI was correlating with the severity of the disease, for example, the degree of splitting of pons, kyphoscoliosis and synergistic convergence were all more marked in the younger girl.

The mechanism of synergistic convergence in our patients is likely to be due to dysfunction of the horizontal gaze mechanism and substitution of convergence for the same.[7,8] Beigi *et al*.[7] and Kohno *et al*.[8] suggested a similar theory in their case reports of patients who had acquired horizontal gaze loss as the result of pontine injury and substituted convergence with miosis for the lost horizontal gaze. This is also in accordance with Leigh and Zee’s model of convergence, suggesting synchronized coexistence of vergence and version system in conjugate eye movement.[9]

Synergistic convergence could be either due to ocular miswiring or horizontal gaze disturbance due to brain stem dysfunction/congenital cleavage of pons. Synergistic convergence in siblings suggests that this may be a variant/part of HGPPS and CCDDs. MRI may be used as a marker to grade the severity of the problem.
